# *shortran*: a pipeline for small RNA-seq data analysis

**DOI:** 10.1093/bioinformatics/bts496

**Published:** 2012-08-22

**Authors:** Vikas Gupta, Katharina Markmann, Christian N. S. Pedersen, Jens Stougaard, Stig U. Andersen

**Affiliations:** ^1^Centre for Carbohydrate Recognition and Signalling, Department of Molecular Biology and Genetics, Aarhus University, Gustav Wieds Vej 10 and ^2^Bioinformatics Research Centre, Aarhus University, C. F. Møllers Allé 8, 8000 Aarhus C, Denmark

## Abstract

**Summary:** High-throughput sequencing currently generates a wealth of small RNA (sRNA) data, making data mining a topical issue. Processing of these large data sets is inherently multidimensional as length, abundance, sequence composition, and genomic location all hold clues to sRNA function. Analysis can be challenging because the formulation and testing of complex hypotheses requires combined use of visualization, annotation and abundance profiling. To allow flexible generation and querying of these disparate types of information, we have developed the *shortran* pipeline for analysis of plant or animal short RNA sequencing data. It comprises nine modules and produces both graphical and MySQL format output.

**Availability:** *shortran* is freely available and can be downloaded from http://users-mb.au.dk/pmgrp/shortran/

**Contact:** vgupta@cs.au.dk or sua@mb.au.dk

**Supplementary information:** Supplementary data are available at *Bioinformatics* online.

## 1 INTRODUCTION

One of the challenges in bioinformatics is to create tools with sufficient flexibility to enable formulation and testing of complex biological hypotheses while remaining technically simple and user friendly. Useful programs for processing small ribonucleic acid (sRNA)-seq data exist, such as the UEA (University of East Anglia) sRNA toolkit (Moxon *et al.*, 2008), wapRNA (Zhao *et al.*, 2011) and Dario (Fasold *et al.*, 2011). However, during our analysis of large-scale sRNA-seq datasets, two issues hampered our progress when using the existing tools. One was restrictions on data upload sizes and parameter adjustment options for web-based tools and the other was a lack of options for easy integration and combined querying of sRNA expression and annotation data. To help address these issues, we developed the *shortran* pipeline for sRNA-seq analysis. It is a command-line Python-based software package consisting of nine modules that can be used independently. The pipeline output includes graphical summaries, text files that can be easily parsed and automatic construction of a MySQL database, which allows simultaneous querying of expression and annotation data. Its features are compared with those of the existing pipelines in Supplementary Table S1. *shortran* is aimed at a broad group of users with only basic bioinformatics training, requiring only little experience with command line and database environments for successful use.

## 2 METHODS

### 2.1 Analysis pipeline summary

**Fig. 1.**
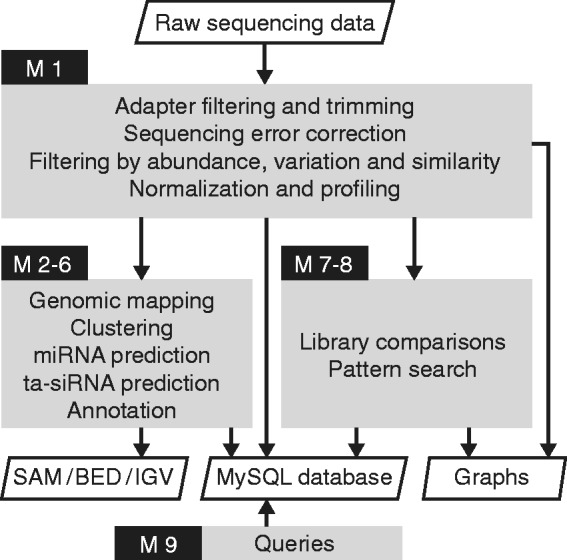
*shortran* flowchart

For maximum flexibility, the pipeline consists of independent modules. They include pre-processing (Module 1), annotation (Modules 2–6), quantitative analysis (Modules 7–8) and database querying (Module 9) (Fig. 1).

### 2.2 Data pre-processing

The data pre-processing implemented in Module 1 enables the user to quickly focus on the most relevant sequences. The first steps are adapter trimming of the raw sequencing files using FASTX (http://hannonlab.cshl.edu/fastx_toolkit/), followed by an optional k-mer correction using ECHO (Kao *et al.*, 2011). Subsequently, read information is summarized in an expression profile table, which is then used as a basis for filtering by abundance and inter-library variation. Finally, Bowtie (Langmead *et al.*, 2009) is used for filtering based on similarity to sequences in a user-provided multi-fasta file. Module 1 also generates a number of graphical data summaries for checking library sizes and length distributions.

### 2.3 Annotation

The mapping and annotation modules feed their results into the MySQL output database, where the annotation data is appended to the profile tables, allowing simultaneous querying of both expression data and annotations. The annotation process starts with mapping filtered reads to a reference genome using Bowtie (Langmead *et al.*, 2009) (Module 2). For convenient visualization, the genomic mapping positions are combined with expression profiles in .igv files (http://www.broadinstitute.org/igv/). As a first step in sRNA categorization, loci characterized by multiple, closely spaced, sRNAs are defined as clusters (Module 3). To predict potential plant and animal miRNAs and plant ta-siRNAs, we have included support for mirDeep-P and mirDeep2 (Friedländer *et al.*, 2012; Yang and Li, 2011) (Module 4), and a modified version of Chen’s algorithm (Chen *et al.*, 2007) (Module 5). As a quality control for miRNA prediction, the results are mapped and compared with conserved miRNAs. Finally, by using Bowtie to map sRNAs against user-supplied sequence files and directly transferring the resulting annotations to the MySQL database, we provide a simple way of adding multiple sets of similarity-based annotations (Module 6).

### 2.4 Comparative quantitative analysis and plotting

To provide an overview of sRNA expression, pairwise scatter plots are generated. Normalized sRNA counts can be compared directly or libraries can be normalized to a reference sample to compare fold changes. In addition, correlation coefficients are calculated (Module 7). The sequence of the first one to three nucleotides of each sRNA is analyzed to identify biases (Module 8).

### 2.5 Database querying

The MySQL database with combined expression and annotation information can be queried using standard SQL syntax, generating a text file output (Module 9).

## 3 APPLICATION

We analyzed 18 sRNA-seq libraries representing the major Arabidopsis root cell types and developmental zones using *shortran* (Supplementary Table S2) (Breakfield *et al.*, 2012). In the original study, the authors focused on miRNAs and briefly mentioned cell-type-specific enrichment of 19-mer sRNAs. Here, we exploit the ease of combining annotation and profiling information using *shortran* to describe the features of this class of sRNAs in detail. The total ∼200 million reads were processed with and without a variation score cutoff (stdev/√average > 20) (Supplementary Fig. S1). There was a large variation in library size, with larger libraries showing higher correlation between replicates (Supplementary Fig. S2 and Table S3). Since the ENDODERMIS2 library was small and showed very poor correlation with all other samples, we ignored it in the subsequent analyses. Application of the variation score cutoff elevated the correlation coefficients (Supplementary Table S3, Supplementary Fig. S3) but did not affect the sequence composition of nucleotides 1–3 (Supplementary Table S4). In accordance with the original study, miRNA prediction by Module 4 identified a number of conserved and putative novel miRNAs that displayed high variation scores (Supplementary Table S5 and Supplementary Table S7 Q1). The graphical data summaries produced by Module 1 showed that the stele and endodermis libraries were strongly enriched for 19-mers compared with the cortex, epidermis and columella samples (Supplementary Figs S4 and S5). To determine the precise nature of the enrichment, we queried the *shortran* MySQL output database (Supplementary Table S6), filtering out 19-mer sRNAs at least three times enriched in stele and endodermis (Supplementary Table S7, Q2). Both construction and querying of the database were computationally light (Supplementary Table S8). All the resulting 69 sRNAs were assigned to the same genomic clusters by Module 3, and Module 6 mapped them all to Glycine transfer ribonucleic acids (tRNAs) with TCC anticodons (Supplementary Table S9 and Fig. S6). Using further queries (Supplementary Table S7 Q3–Q10), we found that the Gly tRNA derived sRNAs fully explained the 19-mer enrichment in the stele and endodermis libraries, and we showed that the enrichment was not limited to the most abundant Gly TTC-associated sRNA (Supplementary Table S10, lines 1–10). This dominant role of Gly tRNAs in sRNA generation agrees with previous observations (Hsieh *et al.*, 2009). Interestingly, the remaining 19-mer tRNA-derived sRNAs were most abundant in columella cells (Supplementary Table S10, lines 11–12). This enrichment was caused exclusively by sRNAs derived from Arg CCT tRNAs (Supplementary Table S7 Q11–12, Table S10 lines 13–16 and Fig. S6), emphasizing the differential cellular expression and potential biological significance tRNA-associated sRNAs.

## 4 CONCLUSION

*Shortran* provides an efficient and user-friendly tool for flexible analysis of sRNA-seq data. Using *shortran*, we were able to quickly generate and combine annotation and profiling information from a complex sRNA-seq experiment. We could then easily mine the data using MySQL queries to identify specific sRNAs for experimental follow-up.

## Supplementary Material

Supplementary Data

Supplementary Data
