# Supplementary material for: shortran: a pipeline for small RNA-seq data analysis
Source: Bioinformatics. 2012 Aug 22;28(20):2698–700. doi: 10.1093/bioinformatics/bts496 (PMC3467745; doi:10.1093/bioinformatics/bts496)
Supplement: Supplementary Data [file supp_28_20_2698__index.html]

shortran: a pipeline for small RNA-seq data analysis — Supplementary Data 

# *shortran*: a pipeline for small RNA-seq data analysis

## Supplementary Data

files

**Files in this Data Supplement:**

- Supplementary Data - pdf file
