# Supplementary material for: shortran: a pipeline for small RNA-seq data analysis
Source: Bioinformatics. 2012 Aug 22;28(20):2698–700. doi: 10.1093/bioinformatics/bts496 (PMC3467745; doi:10.1093/bioinformatics/bts496)
Supplement: Supplementary Data [file supp_bts496_GuptaV_etal_2012_supplementry_file.pdf]

1 SUPPLEMENTARY TABLES

	shortran	UEA tools	wapRNA	DARIO
Adapter trimming	v	v	v	-
Repeat and abundance filtering	v	v	v	-
Multisample normalization and quantification	v	-	-	-
Comparative expression analysis and plotting	v	v[a]	-	-
miRNA prediction	v	v	v	v[b]
Mapping against reference genome	v	v	v	-
Analysis of sRNA-generating loci	v	v	-	v
Annotation by homology	v	-	v	v
Check for 5' sequence composition bias	v	-	-	-
Target gene prediction	-	v	v	-
MySQL-based data integration and querying	v	-	-	-
Web service	-	v	v	v
Downloadable package	v	v	v	-

Supplementary Table 1: Comparison of sRNA-seq analysis pipelines.
[a]: A maximum of two samples. [b]: also predicts other categories of non-coding RNAs (tRNA, snoRNA, etc.)

Sample name	Sample Id	Total raw read counts
STELE1	SRR339951	4021579
STELE2	SRR218092	4710869
ENDODERMIS1	SRR218098	4711441
ENDODERMIS2	SRR339952	2376038
CORTEX1	SRR218096	6313351
CORTEX2	SRR218095	18681349
EPIDERMIS1	SRR218101	7572641
EPIDERMIS2	SRR218102	9796097
COLUMELLA1	SRR218094	11795139
COLUMELLA2	SRR218097	11477250
WR sort 1	SRR218089	21826690
WR sort 2	SRR218090	20311309
WR1	SRR218099	2161398
WR2	SRR218100	4095160
LS1	SRR218085	31147802
LS2	SRR218086	32008086
LS3	SRR218087	30922856
LS4	SRR218088	30898835

Supplementary Table 2: Sequenced libraries.
Data can be found at NCBI Sequence Read Archive (SRA) (http://trace.ncbi.nlm.nih.gov/Traces/sra/sra.cgi) under accession number SRA037191. Sample details are described in Breakfield et al. 2012.

**A** Sample	STELE1	STELE2	ENDODERMIS1	ENDODERMIS2	CORTEX1	CORTEX2	EPIDERMIS1	EPIDERMIS2	COLUMELLA1	COLUMELLA2	WR sort 1	WR sort 2	WR1	WR2	LS1	LS2	LS3
**STELE1**																	
**STELE2**	0.40																
**ENDODERMIS1**	0.42	0.49															
**ENDODERMIS2**	0.12	0.17	0.17														
**CORTEX1**	0.38	0.22	0.49	0.21													
**CORTEX2**	0.13	0.23	0.17	0.05	0.25												
**EPIDERMIS1**	0.33	**0.56**	0.41	0.17	**0.55**	0.32											
**EPIDERMIS2**	0.21	0.33	0.41	0.17	0.35	0.43	0.41										
**COLUMELLA1**	0.18	0.33	0.26	0.15	0.32	**0.59**	0.40	0.44									
**COLUMELLA2**	0.25	0.35	0.30	0.26	0.39	0.27	0.35	0.44	0.37								
**WR sort 1**	0.12	0.18	0.15	0.13	0.24	**0.57**	0.15	0.41	0.35	0.34							
**WR sort 2**	0.15	0.25	0.19	0.10	0.28	**0.65**	0.29	0.45	0.41	0.35	**0.67**						
**WR1**	0.30	0.27	0.32	0.14	0.28	0.09	0.23	0.20	0.17	0.22	0.13	0.14					
**WR2**	0.35	0.44	0.38	0.15	0.45	0.24	0.42	0.33	0.28	0.34	0.24	0.27	0.32				
**LS1**	0.20	0.19	0.19	0.01	0.19	0.28	0.23	0.24	0.26	0.16	0.11	0.21	0.19	0.26			
**LS2**	0.22	0.24	0.23	0.08	0.27	0.14	0.26	0.31	0.15	0.25	0.15	0.20	0.21	0.29	0.44		
**LS3**	0.21	0.18	0.20	0.05	0.24	0.21	0.16	0.26	0.17	0.21	0.19	0.25	0.19	0.23	0.46	**0.56**	
**LS4**	0.22	0.20	0.21	0.09	0.23	0.19	0.17	0.25	0.19	0.25	0.20	0.25	0.23	0.27	0.43	**0.56**	**0.69**

**B** Sample	STELE1	STELE2	ENDODERMIS1	ENDODERMIS2	CORTEX1	CORTEX2	EPIDERMIS1	EPIDERMIS2	COLUMELLA1	COLUMELLA2	WR sort 1	WR sort 2	WR1	WR2	LS1	LS2	LS3
**STELE1**																	
**STELE2**	0.42																
**ENDODERMIS1**	**0.81**	**0.79**															
**ENDODERMIS2**	-0.27	0.13	-0.07														
**CORTEX1**	0.29	**0.79**	0.44	0.18													
**CORTEX2**	0.02	0.30	0.07	-0.01	0.46												
**EPIDERMIS1**	0.27	**0.81**	**0.60**	0.21	**0.90**	0.34											
**EPIDERMIS2**	0.28	**0.64**	**0.52**	0.04	**0.63**	**0.51**	**0.68**										
**COLUMELLA1**	0.00	**0.60**	0.30	0.26	**0.68**	**0.68**	**0.65**	**0.79**									
**COLUMELLA2**	-0.02	**0.63**	0.32	0.31	**0.67**	**0.54**	**0.68**	**0.79**	**0.90**								
**WR sort 1**	0.00	0.17	-0.01	-0.03	0.37	**0.86**	0.23	0.39	0.50	0.39							
**WR sort 2**	0.03	0.24	0.05	-0.07	0.43	**0.87**	0.34	0.49	0.54	0.41	**0.94**						
**WR1**	**0.77**	0.21	**0.63**	-0.24	0.21	0.01	0.16	0.24	0.02	-0.01	-0.01	0.01					
**WR2**	**0.72**	0.48	**0.73**	-0.09	0.46	0.08	0.43	0.42	0.25	0.22	0.02	0.03	**0.78**				
**LS1**	0.41	-0.06	0.21	-0.27	0.08	0.29	0.02	0.01	0.01	-0.11	0.20	0.24	**0.57**	0.44			
**LS2**	0.46	0.08	0.30	-0.15	0.19	0.26	0.15	0.12	0.05	-0.04	0.21	0.23	**0.60**	0.45	**0.90**		
**LS3**	0.50	-0.03	0.26	-0.28	0.06	0.25	0.01	0.09	-0.04	-0.11	0.20	0.20	**0.60**	0.42	**0.90**	**0.91**	
**LS4**	0.45	-0.03	0.25	-0.22	0.09	0.32	0.02	0.16	0.06	-0.02	0.26	0.28	**0.64**	0.49	**0.90**	**0.90**	**0.93**

Supplementary Table 3: Spearman rank correlation coefficients for normalized sRNA counts.

A) Variation score > 0. **B)** Variation score > 20. Values > 0.5 are shown in bold. Tables were generated by Module 7.

variation score > 0		variation score > 20		
**sequence**	**frequency**	**sequence**	**frequency**	**difference**
AA	0.36	AA	0.37	0.01
AC	0.11	AC	0.10	-0.01
AG	0.28	AG	0.27	-0.01
AT	0.24	AT	0.25	0.01
CA	0.31	CA	0.30	0.00
CC	0.13	CC	0.12	-0.01
CG	0.33	CG	0.34	0.01
CT	0.23	CT	0.24	0.00
GA	0.41	GA	0.44	0.04
GC	0.14	GC	0.11	-0.03
GG	0.23	GG	0.22	-0.01
GT	0.23	GT	0.23	0.00
TA	0.20	TA	0.18	-0.02
TC	0.21	TC	0.23	0.02
TG	0.30	TG	0.32	0.02
TT	0.29	TT	0.27	-0.02
AAA	0.37	AAA	0.38	0.01
AAC	0.17	AAC	0.17	0.00
AAG	0.28	AAG	0.26	-0.02
AAT	0.19	AAT	0.19	0.00
ACA	0.35	ACA	0.36	0.01
ACC	0.15	ACC	0.15	0.01
ACG	0.27	ACG	0.25	-0.02
ACT	0.23	ACT	0.23	0.00
AGA	0.46	AGA	0.47	0.01
AGC	0.11	AGC	0.11	0.00
AGG	0.30	AGG	0.29	0.00
AGT	0.13	AGT	0.13	0.00
ATA	0.38	ATA	0.40	0.01
ATC	0.17	ATC	0.17	0.00
ATG	0.23	ATG	0.22	-0.01
ATT	0.22	ATT	0.22	0.00
CAA	0.37	CAA	0.37	0.00
CAC	0.13	CAC	0.14	0.01
CAG	0.29	CAG	0.28	0.00
CAT	0.21	CAT	0.21	0.00
CCA	0.31	CCA	0.31	0.01
CCC	0.14	CCC	0.14	0.00
CCG	0.33	CCG	0.32	-0.01
CCT	0.23	CCT	0.23	0.00
CGA	0.31	CGA	0.31	0.00
CGC	0.13	CGC	0.12	-0.01
CGG	0.42	CGG	0.44	0.03
CGT	0.14	CGT	0.13	-0.01
CTA	0.25	CTA	0.26	0.01
CTC	0.17	CTC	0.16	-0.01
CTG	0.28	CTG	0.25	-0.02
CTT	0.30	CTT	0.33	0.02
GAA	0.36	GAA	0.36	0.00
GAC	0.13	GAC	0.13	0.00
GAG	0.29	GAG	0.28	-0.01
GAT	0.22	GAT	0.22	0.00
GCA	0.27	GCA	0.27	0.00
GCC	0.11	GCC	0.12	0.01
GCG	0.39	GCG	0.36	-0.03
GCT	0.23	GCT	0.25	0.02
GGA	0.26	GGA	0.28	0.02
GGC	0.14	GGC	0.14	0.00
GGG	0.35	GGG	0.34	0.00
GGT	0.25	GGT	0.23	-0.01
GTA	0.26	GTA	0.26	0.00
GTC	0.10	GTC	0.10	0.00
GTG	0.29	GTG	0.27	-0.02
GTT	0.36	GTT	0.37	0.02
TAA	0.33	TAA	0.33	0.01
TAC	0.19	TAC	0.20	0.01
TAG	0.25	TAG	0.25	-0.01
TAT	0.23	TAT	0.22	-0.01
TCA	0.30	TCA	0.32	0.02
TCC	0.17	TCC	0.16	-0.01
TCG	0.23	TCG	0.20	-0.03
TCT	0.30	TCT	0.32	0.02
TGA	0.35	TGA	0.34	0.00
TGC	0.12	TGC	0.12	-0.01
TGG	0.34	TGG	0.36	0.02
TGT	0.19	TGT	0.18	-0.01
TTA	0.22	TTA	0.22	-0.01
TTC	0.17	TTC	0.16	-0.01
TTG	0.22	TTG	0.18	-0.04
TTT	0.39	TTT	0.45	0.06

Supplementary Table 4: Markov chain frequencies of 5’ nucleotides in sRNA sequences. For instance, given TT, the frequency of seeing TTT is 0.39. sRNAs with a variation score > 20 are shown with grey background. The table was generated by Module 8.

Sequence	miRBase.fa	Variation score	Sequence	miRBase.fa	Variation score
TTGACAGAAGATAGAGAGCAC	vvi-miR156i	864.17	AATTGTCAGTATAAATCTTTGATC	-	23.88
TCCCAAATGTAGACAAAGCA	ath-miR158b	650.22	AAGGAGTGGCATGTGAACACA	-	23.71
TGACAGAAGAGAGTGAGCAC	zma-miR156l	647.88	TTGCTTCATAATGGTGAACT	-	23.40
TCGCTTGGTGCAGGTCGGGAA	zma-miR168b	307.34	ACTCATAAGATCGTGACACGT	ath-miR5026	23.19
TCGGACCAGGCTTCATCCCCC	zma-miR166l	287.50	ATCCGAGTTAACGGCTGTTTAAAC	-	23.06
TTCGCTTGCAGAGAGAAATCAC	ath-miR173	247.97	GATCCCCGGCAACGGCGCCA	-	22.79
AAGCTCAGGAGGGATAGCGCC	zma-miR390b	156.52	GGAGCATCATCAAGATTCAC	vvi-miR172d	22.67
TGACAGAAGATAGAGAGCAC	zma-miR156l	141.59	TGGGACTGCCTAAGCTAAAGGCTT	-	22.62
AGCTCTGATACCAAATGATGGAAT	ath-miR829.1	127.30	AGCGGGGTAGAGGAATTGGT	-	22.53
TGACAGAAGAGAGTGAGCACA	zma-miR156j	124.07	TGCCTGGCTCCCTGTATGCCA	zma-miR160g	22.38
TGACAGAAGAGAGTGAGCA	zma-miR156l	120.51	AAATGGATATGAGCAGCCCATGGT	-	22.32
TCCCCAAATGTAGACAAAGCA	-	118.10	AGGCAATAGCTTGAGCAAATA	-	22.25
TCGCTTGGTGCAGGTCGGGAAC	-	115.63	GCGTACAAGGAGTCAAGCA	zma-miR160g	22.12
TCCCAAATGTAGACAAAGC	ath-miR158b	97.89	TCCAATAGGTCGAGCATGTGC	ath-miR862	21.72
GCTCTCTAGCCTTCTGTCATCA	aly-miR157b*	79.52	ATCCCCGGCAACGGCGCCA	-	21.66
AGAATCTTGATGATGCTGCAT	zma-miR172e	71.06	ACTGGTTCAATTGGAGGCGTA	-	21.64
TGAGCCAAGGATGACTTGCCG	vvi-miR169u	66.68	ACGGTATGTTGCGGCTGACTT	-	21.64
TTTGGATTGAAGGGAGCTCTA	zma-miR159k	61.58	TCAATAGATTGGACTATGTAT	ath-miR860	21.62
TCGGACCAGGCTTCATCCC	zma-miR166m	60.98	AGTAACATTGTTGGGGAAAAA	-	21.60
AAACGAACAAAAAACTGATGG	ath-miR837	52.01	TCCGAATGGTGACTGCGTTTT	-	21.32
TCGATAAACCTCTGCATCCAG	vvi-miR162	46.77	AGAGGAAAATATAGAGATCAC	-	21.15
CCCCAAATGTAGACAAAGCA	ath-miR158b	46.51	AAGTGTACGAATCAAACAGTG	-	21.11
TAGCCAAGGATGACTTGCCTGA	vvi-miR169e	44.27	TCGGACCAGGCTTCATCCCC	zma-miR166g	21.01
TCGGACCAGGCTTCATTCCC	zma-miR166i	40.99	AGAGGGGGTGAATGAGAGAGGTTC	-	20.69
TAGCCAAGGATGACTTGCCTG	zma-miR169p	40.31	AGGGTTTAGGGTTTAGGGTTT	-	20.68
AGAAGACCCTTTAAAAACTCTTGT	-	36.34	ACACTTAGTTTTGTACAACAT	-	20.33
CGATCCCCGGCAACGGCGCCA	-	35.10	AGATATTAGTGCGGTTCAATC	gma-miR171	20.27
TCTGGTGTTGAGATAGTTGAC	ath-miR869.2	34.80	AGCTCTGATACCAAATGATGGAA	ath-miR829	20.24
AGAATCTTGATGATGCTGCAG	zma-miR172e	34.17	AGGGTTTAGGGTTTAGGGTTTAGG	-	20.05
AGGCAGTCTCTTTGGCTATC	-	33.84	GACTCATATGGACTTCGGCTA	-	20.03
GGAATCTTGATGATGCTGCAT	zma-miR172e	32.99	TGGATTGGTCAAGGGAAGCGT	ath-miR3440b	19.99
AGCTCAGGAGGGATAGCGCC	zma-miR390b	32.10	TGTTTTGGATCTTAGATACAC	-	19.93
TGAAGCTGCCAGCATGATC	zma-miR167j	31.49	CGTTTGCGGGTGGTAGCGGTT	-	19.67
CGACAGAAGAGAGTGAGCAC	zma-miR156l	30.72	ACAGTGGTCATCTGGTGGGCT	-	19.65
TCGCTTGGTGCAGGTCGGGA	zma-miR168b	25.36	ACATATGATCTGCATCTTTGCATT	-	19.55
ACTTTGAAGCTTTGATTTGAA	aly-miR829*	24.78	TGGTTTCACAGTAGATAGATA	-	19.51

Table continues on next page…

continued from previous page…

Sequence	miRBase.fa	Variation score	Sequence	miRBase.fa	Variation score
GTGGGTGGTAGCGGTTGGTAA	-	19.49	CGAGCTGCTTGATGACTTCATTA	-	15.40
TTAGAGTTTTCTGGATACTTA	ath-miR781	19.37	TCGATCCCCGGCAACGGCGCCA	-	15.40
TGGGTGGTGATCATATAAGAT	ath-miR823	19.29	AGGGTTTAGGGTTTAGGGTT	-	15.39
CGACAGAAGAGAGTGAGCACA	zma-miR156j	19.22	TTGTGCGGTTCAAATAGTAAC	-	15.37
TAATCTGCATCCTGAGGTTTA	vvi-miR2111	19.18	AAAGAGGGCTTATATTACT	-	15.31
GCAAGTTGACCTTGGCTCT	aly-miR169g	18.53	AACAAGTAGACATATAAAAACAT	-	15.15
TCATTGAGTGCATCGTTGATG	zma-miR397b	18.20	TGACATGGGACTGCCTAAGCTA	ath-miR848	15.09
CTGAAGTGTTTGGGGGGACTC	zma-miR395	18.14	GGAGGATATAATTAATCAGA	-	14.93
AGGCAGTCTCCTTGGCTATC	-	18.11	TGGGTGGTGATCATATAAGA	ath-miR823	14.81
ACAAAGTTTTATACTGACAAT	aly-miR4245	18.06	GCGTACAGAGTAGTCAAGCA	aly-miR160b*	14.68
TCATGGGTTGAGTTGAGTTGA	-	18.01	GAGATTGTTTACCGGCCATTTTA	-	14.64
TGGAGGCAGCGGTTCATCGATC	csi-miR162*	17.55	TACTATTTGAATCGTACTGC	-	14.60
ATCCTTATTGATGATCTCTTAACA	-	17.55	TCCCAAATGTAGACAAAGCAAT	-	14.56
ATGCGATTGAGAGCAACAAGACAT	-	17.34	GGCAGTCTCCTTGGCTATTC	-	14.56
TTGATTCGTACACTTGGATT	-	17.29	AGATAAAAATAGAGATGAG	-	14.54
TTCGCAGGAGAGATAGCGCCA	ath-miR391	17.29	AGATAAAAATAGAGATGAGT	-	14.50
TCAGGTATGATTGACTTCAAA	ath-miR864	17.10	TGGAGAAGCAGGGCACGTGC	zma-miR164h	14.49
CTGAAGTGTTTGGGGGAACTC	zma-miR395p	16.86	ATTTGGCCGTTTAATACCT	-	14.37
TGATTGGAAATTTCGTTGACT	ath-miR779	16.81	TGCGAGGGAATTGCGACTTTTTA	-	14.32
GCCGGTGGTCGCGAGAGGGAT	-	16.60	TGGAAGATGCTTTGGGATTTATT	-	14.24
TATGAGAGTATTATAAGTCAC	ath-miR400	16.50	CAAGATTTACAAGGATGGTTTTA	-	14.19
GGGTTGATATGAGAACACACG	aly-miR398b	16.24	ATAATCGCCTTAGAAAACGTCATT	-	14.00
TCGGACCAGGCTTCATCCCCCT	ctr-miR166	16.11	TGCGATTGAGAGCAACAAGAC	-	13.99
GAATTAGGGTCAGTCACTTT	-	16.06	GGCCGGTGGTCGCGAGAGGGATT	-	13.97
TTGGTAGTGGATAAGGGGGCA	-	16.05	AGATAAGCGCCTTAGTTCTGA	aly-miR852	13.94
TAGGGTTTAGGGTTTAGGGTTTA	-	15.98	AGAGATAATTGGTCTTGGTTTCA	-	13.88
ATTTGATTATGAGTTGAGTT	-	15.94	TGGGACTGCCTAAGCTAAAGGCT	-	13.83
TCCGGATCTGTAGGATCTACGGTG	-	15.90	AGATTTGAAGATTCAAGGACATC	-	13.79
ATGGAGAGAGTTGGCCAACT	-	15.87	GCTCTCGTAGCTCAGTTGGT	-	13.76
AAGATAAGCGCCTTAGTTCT	aly-miR852*	15.86	TTCGGGTCGATTCGGTTATTTTA	-	13.65
GCAGCACCATTAAGATTCACAT	-	15.86	TCGATTTGGGGATTTAGGGTTTA	-	13.54
TGGAGGCAGCGGTTCATCGA	csi-miR162*	15.80	TGGGGATGGAGTTAGATGGCTTA	-	13.41
TGCCAAAGGAGAGTTGCCCT	zma-miR399j	15.79	CGTACAAGGAGTCAAGCATG	zma-miR160g*	13.20
ATCACATACGGACATACACGAAC	-	15.67	AACTGACAGAAGAGAGTGAGCAC	-	13.10
TGTATAAAATATGGGTTACT	-	15.55	GCAGCACCATTAAGATTCAC	bra-miR172b*	13.06
TGCCAAAGGAGATTTGCCCGG	zma-miR399j	15.47	TAAGAACTCAAGCTAAGATCCCA	-	12.87

Table continues on next page…

continued from previous page…

Sequence	miRBase.fa	Variation score	Sequence	miRBase.fa	Variation score
TGACAGAAGAAAGAGAGCAC	zma-miR156j	12.87	GGGCAAGATCACCATTGGCAGA	aly-miR399	8.75
GATAAGGAGACATTTTGGAGCAA	-	12.73	GGGTAAATATCCAGATTTTCGG	-	8.73
AGGGACAGTAGATGTCACAGGTT	-	12.66	TACGCATTGAGTTTCGTTGCTT	ath-miR777	8.66
GTTGCGGCAACTAAACGAACATA	-	12.64	AGAAGATACAAAGACAAAGACT	-	8.54
GTATGAGGAGCCATGCATA	bra-miR160a*	12.60	TGACAGAAGAGAGTGAGCACAC	-	8.53
TGAAGCTGCCAGCATGATCT	zma-miR167j	12.58	AGGGTTTAGGGTTTAGGGTTTA	-	8.35
GTTTGCGGGTGGTAGCGGTT	-	12.40	TCAGCTAAGATCCGGACTACAA	-	8.13
TTTGCTTCCAGCTTTTGTCTCC	-	12.27	TCATGGTCAGATCCGTCATCCC	-	7.47
AGCCAAGGATGACTTGCCGG	zma-miR169r	12.26	AACTCATAAGATCGTGACACGT	-	7.34
AGAGTAGGGTTGGAGATGC	-	12.14	AAGTGTACGAATCAAACAGTGA	-	7.24
AGTGAAAGATATTGTAGTGTATA	-	12.11	TCAGCTGAGCTTTCTCGTCATC	-	7.00
ATCCTTATTGATGATCTCTTAAC	-	11.89			
AAAGCTCAGGAGGGATAGCGCC	-	11.80			
GCAAGTTGACCTTGGCTCTGTT	-	11.74			
AACTTTGGTTTCGTCGGACGACA	-	11.69			
TTTGGATTGAAGGGAGCTC	zma-miR159d*	11.43			
AGAATCTTGATGATGCTGCA	zma-miR172e	11.43			
CAGCCAAGGATGACTTGCCG	zma-miR169r	11.41			
GCGTATGAGGAGCCATGCAT	bra-miR160a*	11.22			
CAGGGAACAAGCAGAGCATG	aly-miR408*	10.97			
GCGGCGACGAAACGAACAGACTA	-	10.67			
TCTGGGATGAATTTGGATC	-	10.56			
AGCTCTGATACCAAATGATGGA	ath-miR829	10.17			
GGCAGTCTCCTTGGCTATT	aly-miR169n*	10.10			
AAAGCTATACAACAGGTGGACG	-	10.09			
TTGTTTGTTGTACTCGGTCTAG	-	9.82			
AAGGGATTTTAAAAGGGTTTTA	-	9.75			
ACAAGGGATTTTAAAAGGGTTT	-	9.66			
ATGAAATTTTGTGGTCTCGGCA	-	9.65			
GGGCGAATACTCCTATGGCAGA	aly-miR399d*	9.58			
AGAAAGAGCTGTATAAGAAAGC	-	9.54			
AGGGAACTCTATTTAGACGCA	-	9.10			
AGGGTTAAATGAGTTATGGGTT	-	9.07			
AAGCGATGTGGGATATTGTACA	-	9.04			
TAGCCAAGGATGACTTGCCTGT	vvi-miR169e	9.03			
AGCCAAGGATGACTTGCCGA	zma-miR169r	9.01			

Supplementary Table 5: miRNAs predicted with miRDeep-P (Module 4).
In the miRBase.fa column, the top miRBase (v17.0) hit is shown. sRNAs with a variation score > 20 are shown with grey background. Predicted miRNAs with a high variation score frequently map to conserved miRNA sequences.

Column Name	Description
Sequence	sRNA sequence
STELE1	
STELE2	
ENDODERMIS1	
ENDODERMIS2	
CORTEX1	Raw sRNA counts
CORTEX2	
EPIDERMIS1	
EPIDERMIS2	
COLUMELLA1	
COLUMELLA2	
WR1	
WR2	
WR_sort_1	
WR_sort_2	
LS1	
LS2	
LS3	
LS4	
sum	Sum of the raw counts
STELE1_norm	
STELE2_norm	
ENDODERMIS1_norm	
ENDODERMIS2_norm	
CORTEX1_norm	
CORTEX2_norm	
EPIDERMIS1_norm	Normalized sRNA counts
EPIDERMIS2_norm	
COLUMELLA1_norm	
COLUMELLA2_norm	
WR1_norm	
WR2_norm	
WR_sort_1_norm	
WR_sort_2_norm	
LS1_norm	
LS2_norm	
LS3_norm	
LS4_norm	
sum_norm	Sum of the normalized counts
score	Variation score (stdev/sqrt(average))
sequence_length	
miRNA_prediction_mirdeep2	
miRNA_prediction_mirdeepP	
ta-siRNAprediction	
genomic_region	
TAIR10_chr_all.fa	
miRBase.fa	Annotation
clusters.fa	
ath_tRNA.fa	
ath_tas.fa	
TIGR_Brassicaceae_Repeats.v2_0_0.fa	

Supplementary Table 6: MySQL table summary.
miRNA_prediction and ta-siRNA_prediction: results of prediction algorithms. genomic_region: sRNAs are categorized as exonic, intronic, overlapping exon/intron boundaries, intergenic or not mapped by comparing mapping information with genomic feature annotation. All columns ending on .fa. Non-matching sRNAs are assigned “0”, while the fasta header and the position coordinates are given for matching reads. TAIR10_chr_all.fa: *Arabidopsis thaliana* genome (TAIR10). miRBase.fa: miRNA sequences annotated in miRBase. clusters.fa: reads map as part of the clusters called by Module 3. ath_tRNA.fa: tRNA sequences from *Arabidopsis thaliana*. ath_tas.fa: TAS loci in *Arabidopsis thaliana*. TIGR_ Brassicaceae _Repeats.v2_0_0.fa: Brassicaceae family repeats from the TIGR database.

**Purpose**	**Syntax**
Q1: Retrieve variation scores and miRBase annotations for predicted miRNAs. Suppl. Table 5	SELECT<br>`#Sequence`,<br>` miRBase.fa`,<br>`score`<br>FROM<br>`ath_2012-06-24_profile_19_24_sorted.cut-30_score_0`<br>WHERE ` miRNA_prediction` = "predicted as miRNA";
Q2: Retrieve annotation and profiling information for 19-mer sRNAs enriched in stele and endodermis cell types. Suppl. Table 9.	SELECT<br>`#Sequence`,`score`,`read_size`, STELE1_norm,STELE2_norm, ENDODERMIS1_norm, CORTEX1_norm,CORTEX2_norm, COLUMELLA1_norm,COLUMELLA2_norm, EPIDERMIS1_norm, EPIDERMIS2_norm, `miRBase.fa_T`,`miRNA_prediction_mirdeepP`,`clusters.fa`,`ta-siRNAprediction`, genomic_region,`ath_tas.fa`,`ath_tRNA.fa`<br>FROM<br>`ath_2012-06-24_profile_19_24_sorted.cut-30_score_0`<br>WHERE<br>(STELE1_norm + STELE2_norm)/2 > 20 AND<br>abs(STELE1_norm - STELE2_norm)/(STELE1_norm + STELE2_norm) < 0.5 AND<br>(CORTEX1_norm + CORTEX2_norm)/2 < (STELE1_norm + STELE2_norm)/2/3 AND<br>(COLUMELLA1_norm + COLUMELLA2_norm)/2 < (STELE1_norm + STELE2_norm)/2/3 AND<br>(EPIDERMIS1_norm + EPIDERMIS2_norm)/2 < (STELE1_norm + STELE2_norm)/2/3 AND<br>(CORTEX1_norm + CORTEX2_norm)/2 < ENDODERMIS1_norm/3 AND<br>(COLUMELLA1_norm + COLUMELLA2_norm)/2 < ENDODERMIS1_norm/3 AND<br>(EPIDERMIS1_norm + EPIDERMIS2_norm)/2 < ENDODERMIS1_norm/3 AND<br>read_size = 19<br>ORDER BY (STELE1_norm + STELE2_norm)/2 DESC<br>LIMIT 10;
Q3: Summarize expression of all tRNA-derived sRNAs. Suppl. Table 10, line 1.	SELECT<br>sum((COLUMELLA1_norm+COLUMELLA2_norm)/2),<br>sum((STELE1_norm+STELE2_norm)/2),<br>sum((CORTEX1_norm+CORTEX2_norm)/2), sum(ENDODERMIS1_norm),<br>sum((EPIDERMIS1_norm+EPIDERMIS2_norm)/2)<br>FROM<br>`ath_2012-06-24_profile_19_24_sorted.cut-30_score_0`<br>WHERE<br>`ath_tRNA.fa` != 0;
Q4: Summarize expression of 19-mer tRNA-derived sequences. Suppl. Table 10, line 2.	SELECT<br>sum((COLUMELLA1_norm+COLUMELLA2_norm)/2),<br>sum((STELE1_norm+STELE2_norm)/2),<br>sum((CORTEX1_norm+CORTEX2_norm)/2), sum(ENDODERMIS1_norm),<br>sum((EPIDERMIS1_norm+EPIDERMIS2_norm)/2)<br>FROM<br>`ath_2012-06-24_profile_19_24_sorted.cut-30_score_0`<br>WHERE<br>`ath_tRNA.fa` != 0 AND<br>read_size = 19;

Q5: Summarize expression of Gly TTC tRNA-derived sequences. Suppl. Table 10, line 3.	SELECT<br>sum((COLUMELLA1_norm+COLUMELLA2_norm)/2),<br>sum((STELE1_norm+STELE2_norm)/2),<br>sum((CORTEX1_norm+CORTEX2_norm)/2), sum(ENDODERMIS1_norm),<br>sum((EPIDERMIS1_norm+EPIDERMIS2_norm)/2)<br>FROM<br>`ath_2012-06-24_profile_19_24_sorted.cut-30_score_0`<br>WHERE<br>`ath_tRNA.fa` LIKE "%trna51-GlyTCC%";
Q6: Summarize expression of Gly TTC tRNA-derived 19-mer sequences. Suppl. Table 10, line 4.	SELECT<br>sum((COLUMELLA1_norm+COLUMELLA2_norm)/2),<br>sum((STELE1_norm+STELE2_norm)/2),<br>sum((CORTEX1_norm+CORTEX2_norm)/2), sum(ENDODERMIS1_norm),<br>sum((EPIDERMIS1_norm+EPIDERMIS2_norm)/2)<br>FROM<br>`ath_2012-06-24_profile_19_24_sorted.cut-30_score_0`<br>WHERE<br>`ath_tRNA.fa` LIKE "%trna51-GlyTCC%" AND<br>read_size = 19;
Q7-Q10: Repeat analysis described in Q3-6 excluding the most abundant Gly TTC tRNA-derived sRNA. Suppl. Table 10, lines 5-10.	# Like queries Q3-6 with the added condition that:<br>`#Sequence` != "GCGTCTGTAGTCCAACGGT"
Q11: Summarize expression of non Gly TTC tRNA-derived sRNAs grouped by tRNA. Suppl. Table 10, lines 13 and 15.	SELECT<br>substring(`ath_tRNA.fa`,24,19), sum((STELE1_norm+STELE2_norm)/2),<br>sum(ENDODERMIS1_norm), sum((CORTEX1_norm+CORTEX2_norm)/2),<br>sum((COLUMELLA1_norm+COLUMELLA2_norm)/2),<br>sum((EPIDERMIS1_norm+EPIDERMIS2_norm)/2)<br>FROM<br>`ath_2012-06-24_profile_19_24_sorted.cut-30_score_0`<br>WHERE<br>`ath_tRNA.fa` != 0 AND<br>`ath_tRNA.fa` NOT LIKE "% trna51-GlyTCC %"<br>GROUP BY substring(`ath_tRNA.fa`,24,19)<br>ORDER BY sum((COLUMELLA1_norm+COLUMELLA2_norm)/2) DESC<br>LIMIT 2;
Q12: Summarize expression of non Gly TTC tRNA-derived sRNAs grouped by tRNA, 19-mers only. Suppl. Table 10, lines 14 and 16.	SELECT<br>substring(`ath_tRNA.fa`,24,19), sum((STELE1_norm+STELE2_norm)/2),<br>sum(ENDODERMIS1_norm), sum((CORTEX1_norm+CORTEX2_norm)/2),<br>sum((COLUMELLA1_norm+COLUMELLA2_norm)/2),<br>sum((EPIDERMIS1_norm+EPIDERMIS2_norm)/2)<br>FROM<br>`ath_2012-06-24_profile_19_24_sorted.cut-30_score_0`<br>WHERE<br>`ath_tRNA.fa` != 0 AND<br>`ath_tRNA.fa` NOT LIKE "% trna51-GlyTCC %" AND<br>read_size = 19<br>GROUP BY substring(`ath_tRNA.fa`,24,19)<br>ORDER BY sum((COLUMELLA1_norm+COLUMELLA2_norm)/2) DESC<br>LIMIT 2;

Supplementary Table 7: Examples of MySQL queries used to generate Suppl. Tables 5, 9 and 10.

Module Number	Name	Time in seconds	CPU %	Memory %
**1.1**	Adapter filtering/Trimming	6,616	10.7	0.9
**1.1**	Error correction	100,167	93.7	1.9
**1.1**	Size splitting	1,516	65.7	1.3
**1.2**	Making profile tables	1,832	55.3	3.2
**1.3**	Adding to MySQL	35	0.9	1.1
**2**	Genomic mapping	341	136.2	9.5
**3.1**	Cluster prediction	304	123.5	0.7
**3.2**	Cluster genomic region prediction	6,937	101.1	2.3
**4.1**	miRNA predictions-mirDeep2	12,286	99.3	5.6
**4.1**	miRNA predictions-mirDeepP	164,049	9.4	5.6
**4.2**	miRBase mapping	3	2.7	2.3
**5**	ta-siRNA prediction	1,397	90.3	1.2
**6**	Annotation to MySQL (mapping)	232	20.3	0.4
**6**	Annotation to MySQL (database)	11	0.6	0.5
**7**	Expression plotting	2,209	98.5	2.3
**8.1**	Find pattern	4	101.2	1
**8.2**	Map pattern	155	79.5	2.3
**9**	Querying	1	102.9	0.3

Supplementary Table 8: Time, CPU and Memory usage summary for all modules.
shortran was run on an Ubuntu 12.04 LTS (GNU/Linux 3.2.0-25-generic x86_64) system with eight Intel(R) Xeon(R) CPU W3540 2.93GHz cores and 20 GB RAM.

#Sequence	score	size	STE1	STE2	END1	CRT1	CRT2	CLM1	CLM2	EPI1	EPI2
GCGTCTGTAGTCCAACGGT	903	19	647,169	336,284	848,694	56,593	3,553	27,310	59,746	104,767	167,124
GCGTCTGTAGTCCCACGGT	138	19	5,678	3,833	11,581	102	2	60	283	122	354
GCGTCTGTAGTCCAACGGC	122	19	5,143	3,265	8,479	145	3	19	24	305	133
GCGTCTGTAGTCCAACGGG	101	19	6,049	2,029	7,099	251	3	99	297	721	633
GCGTCTGTAGTCCAAAGGT	139	19	3,401	2,811	7,552	120	1	71	172	152	3,230
GCGTCTGTAGTCCACCGGT	110	19	3,410	2,451	6,857	55	3	14	45	101	85
GCGTCTGTAGTCCAACGGA	92	19	1,731	2,068	5,048	37	3	19	71	80	250
GCGTCTGTAGTCCAACCGT	63	19	1,736	1,063	1,413	27	4	6	44	35	99
GCGTCTGTAGTCAAACGGT	72	19	1,204	1,337	3,110	56	1	6	12	109	187
GCGTCTGTAGTCCAACGCT	48	19	1,108	483	1,035	24	1	4	6	32	22

Supplementary Table 9: Top ten 19-mer sRNAs enriched in stele and endodermis cell types. Sequences were sorted by average expression in the stele cell type. STE: stele, END: endodermis, CRT: cortex, CLM: columella, EPI: epidermis. All sequences were assigned to 12 clusters by Module 3 (12;lcl|Chr5:3578295-3578374_54,lcl|Chr4:10325965-10326044_54,lcl|Chr3:16427041-16427120_54,lcl|Chr2:12471773-12471852_54,lcl|Chr2:11864151-11864233_57,lcl|Chr1:4285887-4285966_54,lcl|Chr5:3617055-3617134_4,lcl|Chr5:23957427-23957511_4,lcl|Chr5:23953940-23954018_3,lcl|Chr5:18558843-18558921_3,lcl|Chr3:17149749-17149828_4,lcl|Chr1:2588522-2588601_4) and to 12 tRNA sequences by Module 6 (12;chr5.trna51-GlyTCC_1,chr5.trna50-GlyTCC_1,chr5.trna34-GlyTCC_1,chr5.trna13-GlyTCC_1,chr5.trna105-GlyTCC_1,chr4.trna63-GlyTCC_1,chr3.trna74-GlyTCC_1,chr3.trna35-GlyTCC_1,chr2.trna75-GlyTCC_1,chr2.trna74-GlyTCC_1,chr1.trna232-GlyTCC_1,chr1.trna14-GlyTCC_1).

		Stele	Endodermis	Cortex	Columella	Epidermis
1	All tRNAs	560,189	930,771	56,567	65,106	176,838
2	tRNAs, 19-mers only	545,089	925,675	37,016	58,146	151,144
3	All Gly TTC tRNAs	551,986	927,914	45,978	48,895	161,982
4	Gly TTC tRNAs, 19-mers only	542,162	924,126	32,328	45,527	144,262
5	All 19-mers	558,705	931,074	70,870	132,424	179,177
6	tRNAs without most abundant Gly TTC	68,462	82,077	26,494	21,578	40,892
7	tRNAs, 19-mers only without most abundant Gly TTC	53,363	76,981	6,943	14,617	15,198
8	Gly TTC tRNAs without most abundant Gly TTC	60,259	79,220	15,905	5,367	26,036
9	Gly TTC tRNAs, 19-mers only, without most abundant Gly TTC	50,436	75,432	2,255	1,999	8,316
10	19-mers, without most abundant Gly TTC	66,979	82,381	40,797	88,896	43,231
11	tRNAs except Gly TTC	8,203	2,857	10,589	16,211	14,856
12	19-mer tRNAs except Gly TTC	2,927	1,548	4,688	12,619	6,882
13	All Arg CCT tRNAs	840	512	1,904	9,708	2,648
14	Arg CCT tRNAs, 19-mers only	749	472	1,809	9,386	2,478
15	All Asp GTC tRNAs	3,224	719	2,954	2,126	4,802
16	Asp GTC tRNAs, 19-mers only	641	175	1,017	1,322	1,739

Supplementary Table 10: Summary of expression counts for 19-mers and tRNA-derived sRNAs. In lines 13-16 only the top two expressed tRNA groups are shown, since they account for the majority of the reads.

2 SUPPLEMENTARY FIGURES

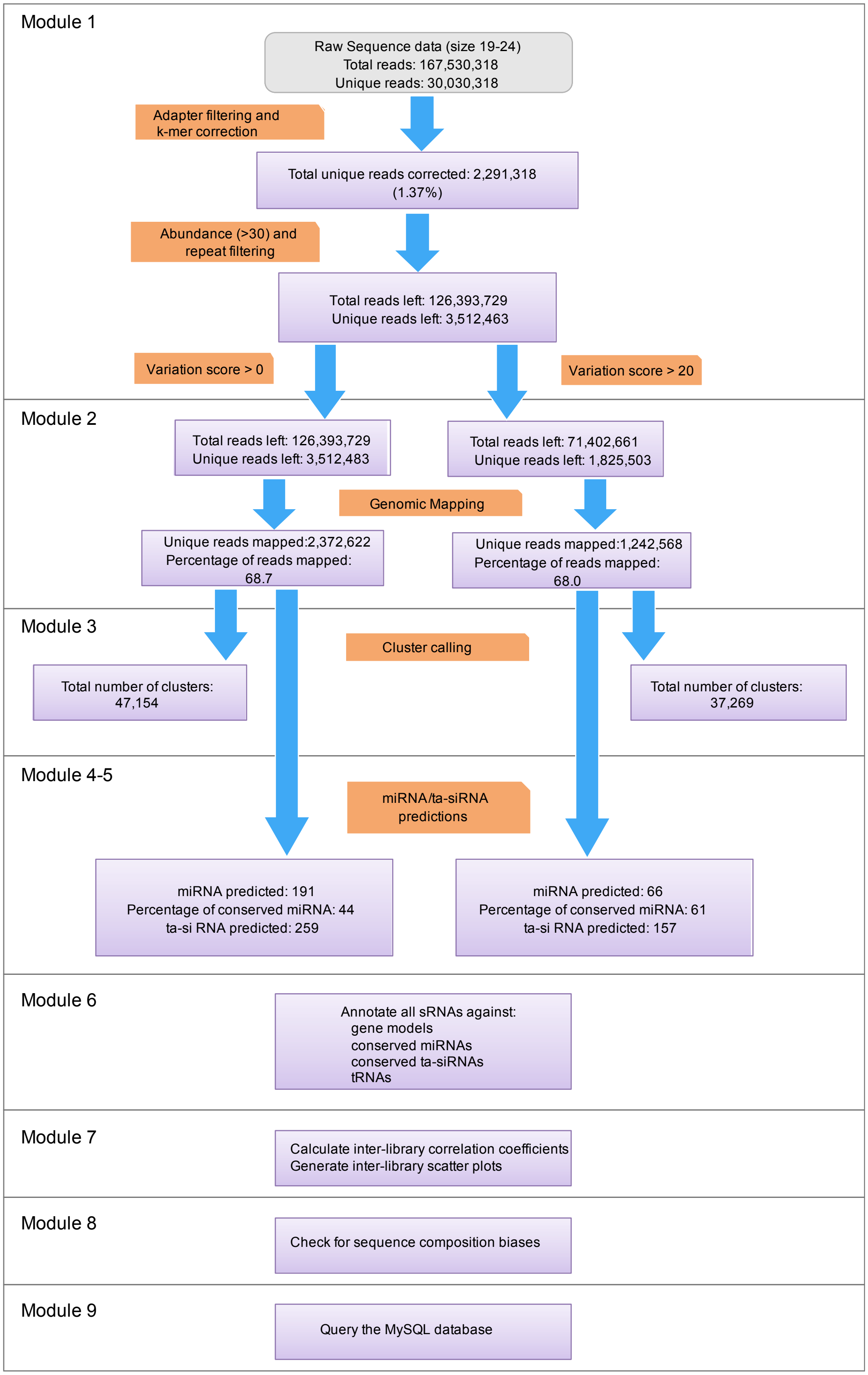

Supplementary Figure 1: Data processing overview.

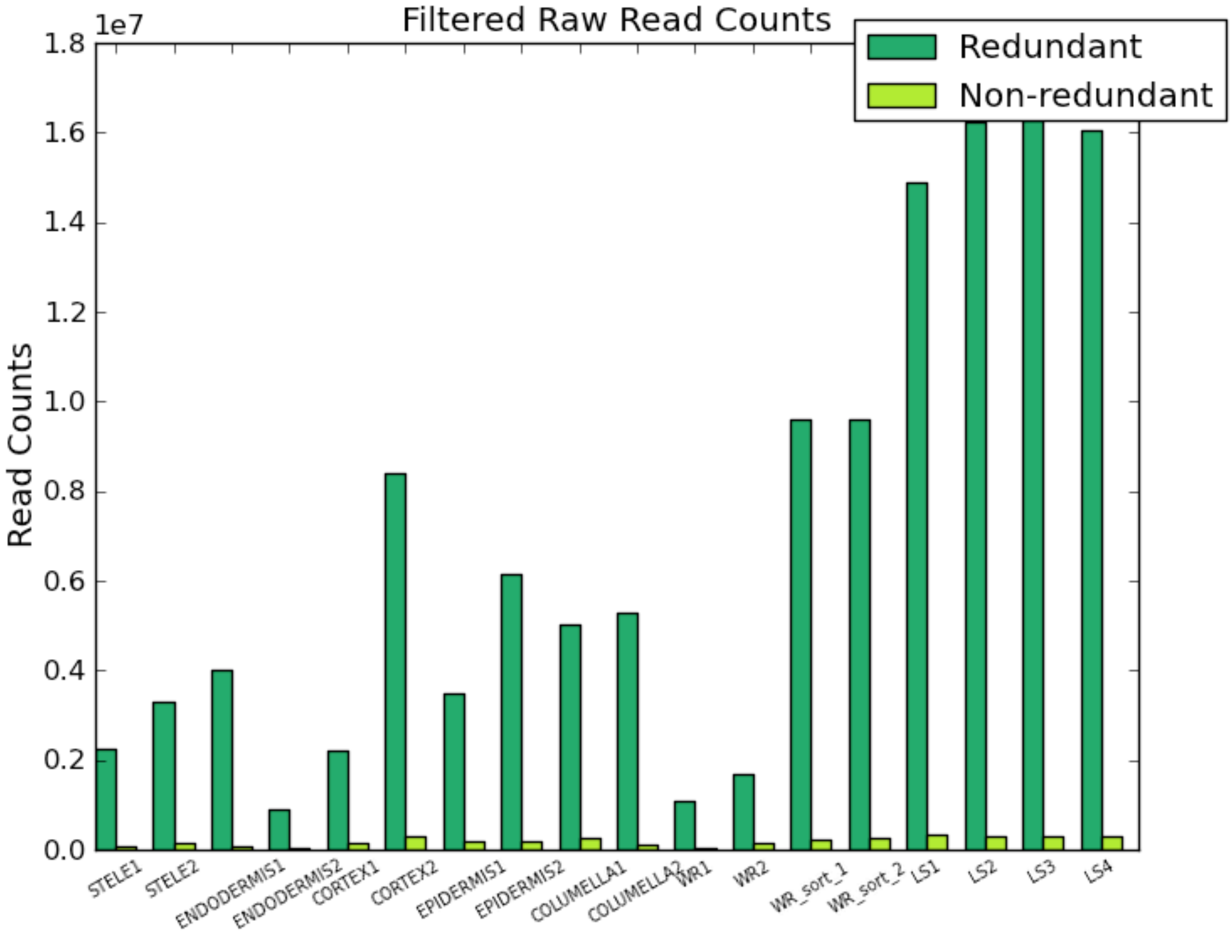

Supplementary Figure 2: Library size overview.
Redundant counts represent the total number of reads in each library. The Non-redundant counts represent the total number of unique sequences within each library. This figure is generated by Module 1 to allow checking for consistent library sizes.

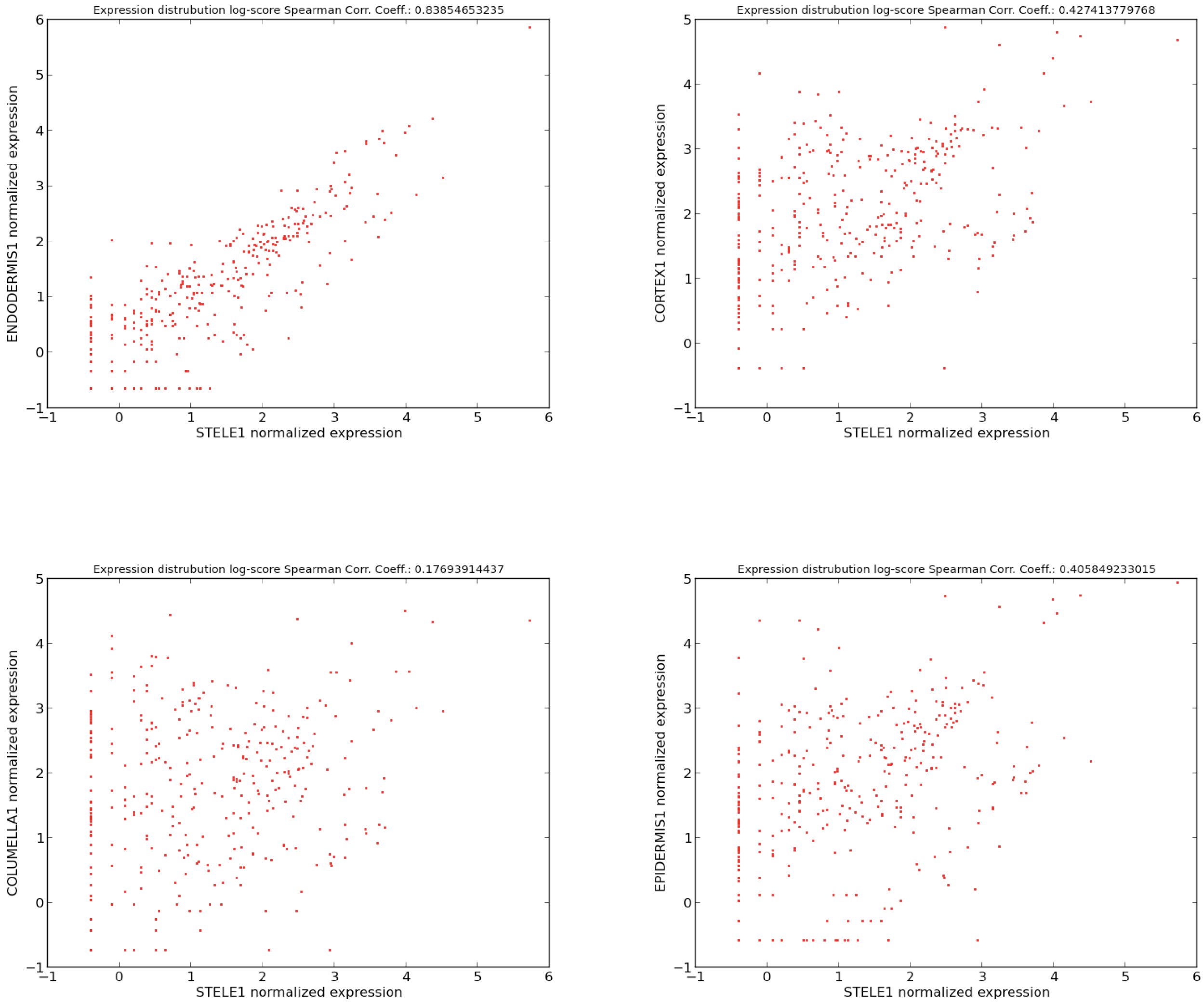

Figure continues on next page...

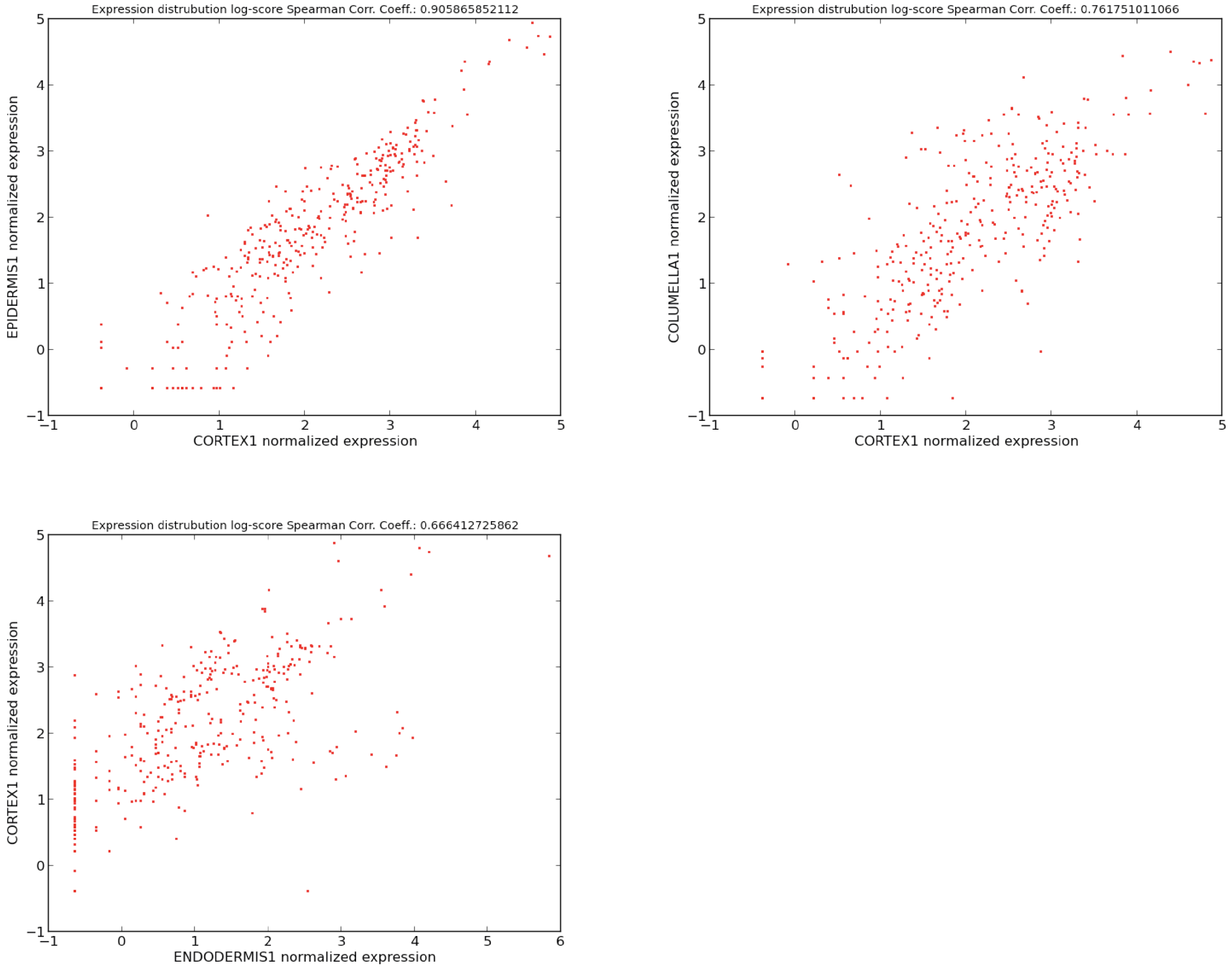

Supplementary Figure 3: Inter-library comparisons of normalized log counts. Size fractions 19-24 are all plotted. A variation score cutoff of 20 was applied.

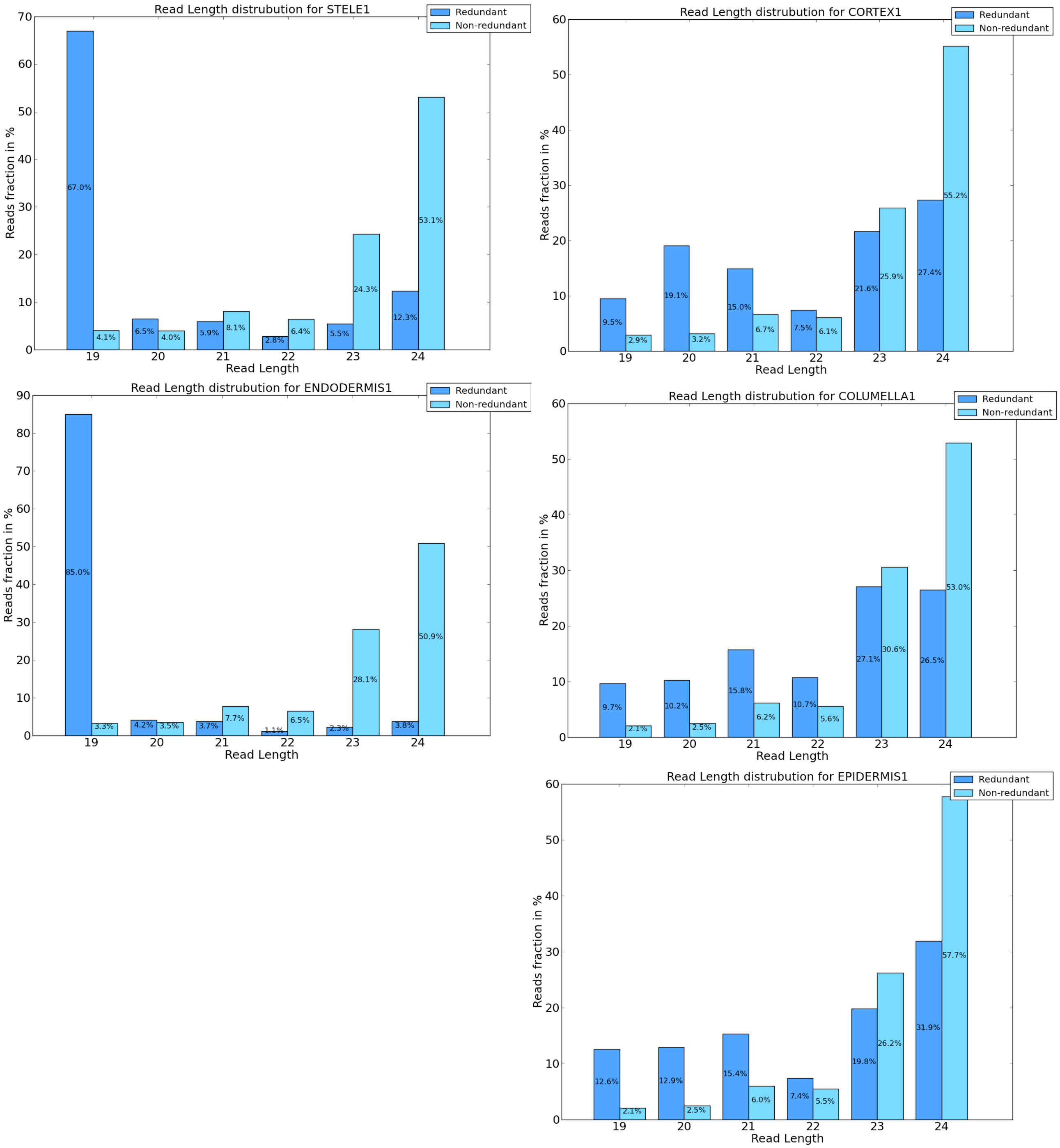

Supplementary Figure 4: Size fraction distribution for all root cell types.

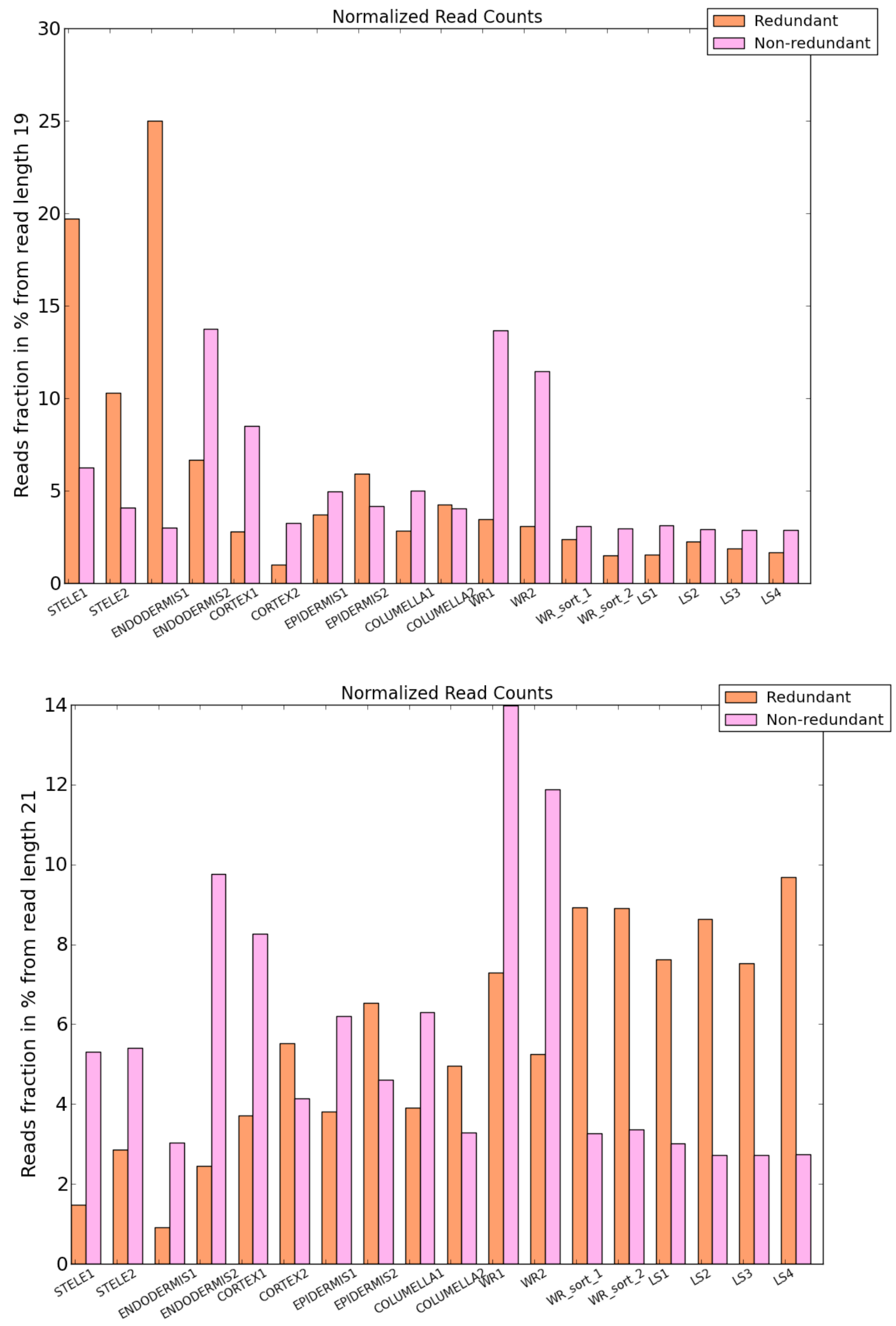

Supplementary Figure 5: 19 and 21 size sequence distribution for all libraries.

A

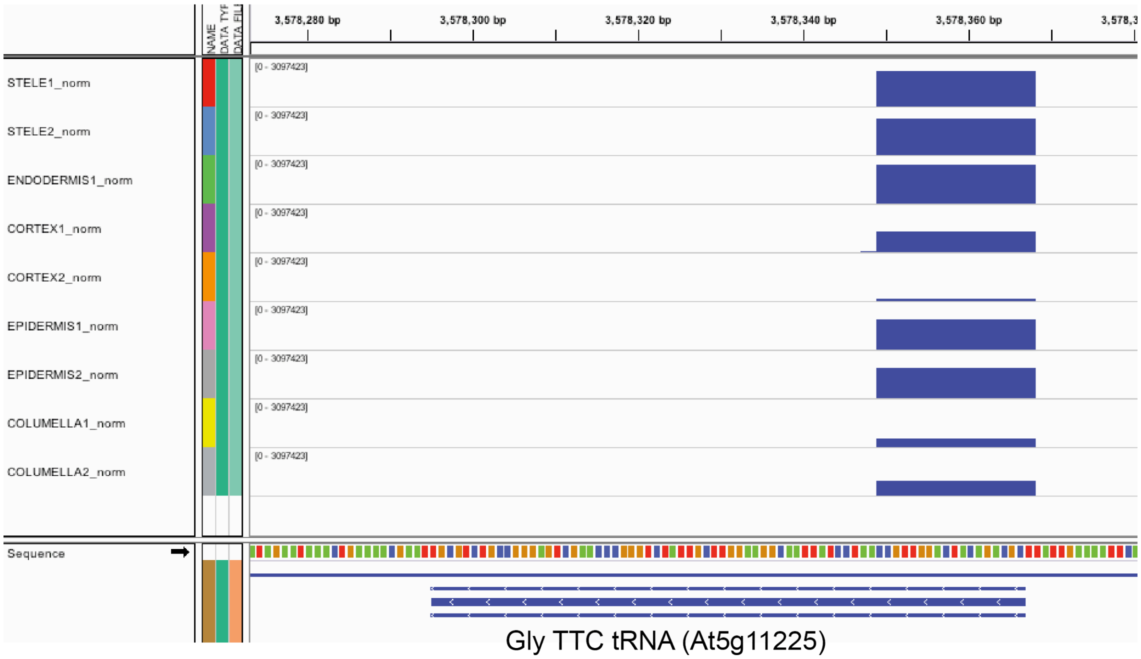

B

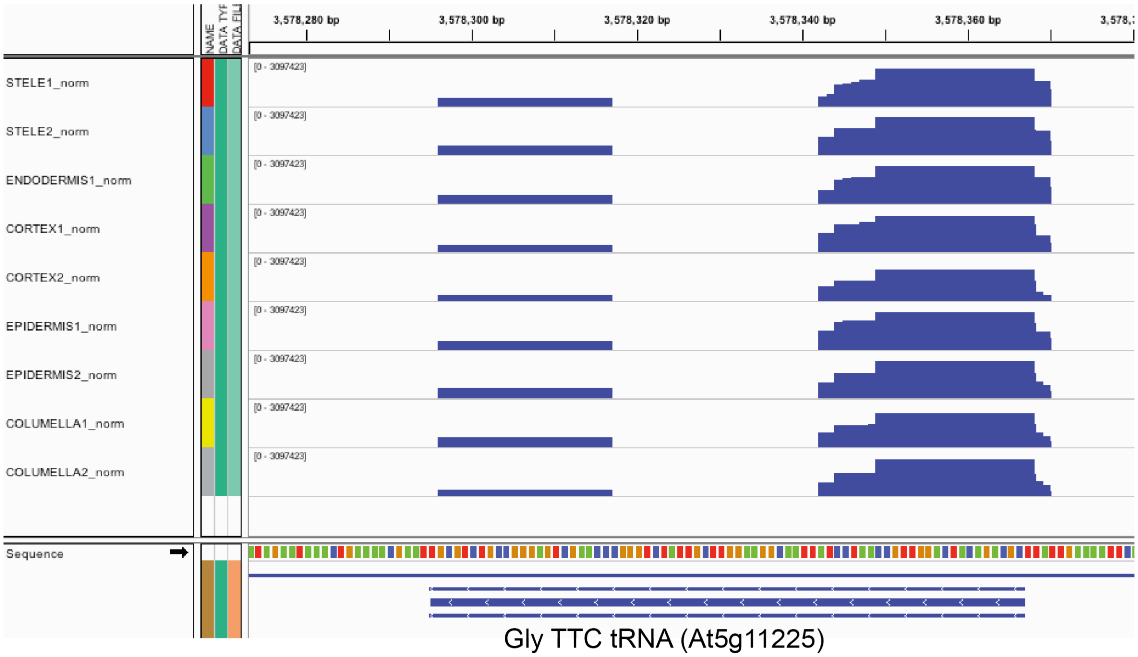

Figure continues on next page...

C

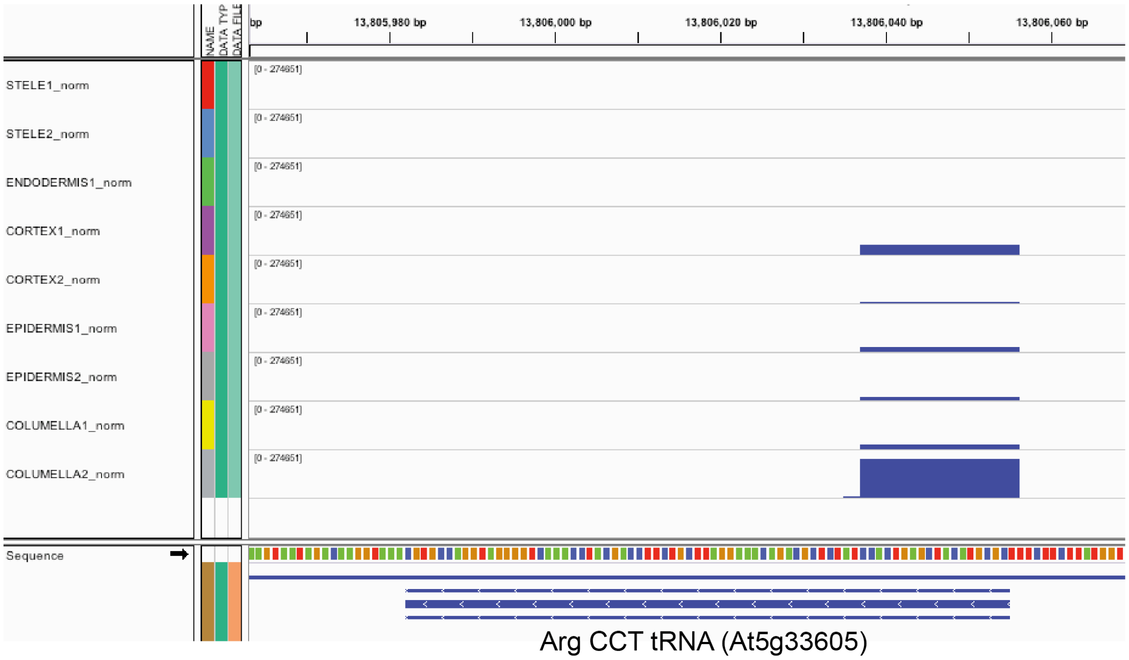

D

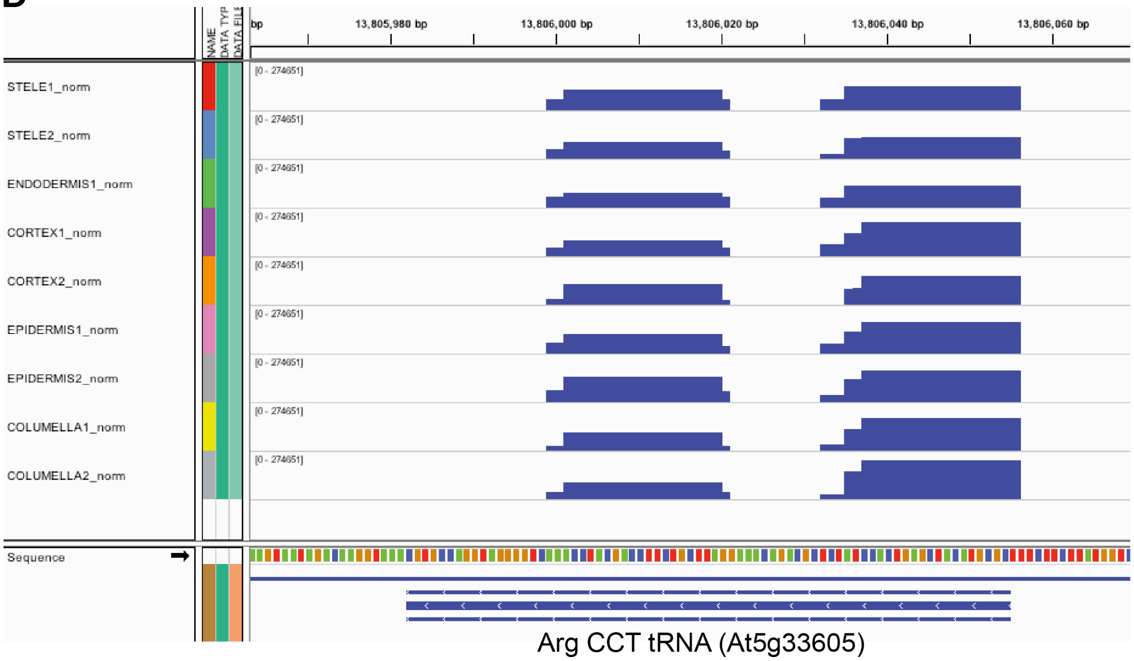

Supplementary Figure 6: IGV visualization of sRNA generating tRNA loci.
A-B) Gly TTC tRNA locus. **C-D**) Arg CCT tRNA locus. **A,C**) Linear scale. **B,D)** Logarithmic scale. Both tRNA loci generate a set of highly abundant sRNAs and one much less abundant set, reminiscent of the miRNA and miRNA* generated from miRNA loci.
